# Citizen Science, Education, and Learning: Challenges and Opportunities

**DOI:** 10.3389/fsoc.2020.613814

**Published:** 2020-12-02

**Authors:** Joseph Roche, Laura Bell, Cecília Galvão, Yaela N. Golumbic, Laure Kloetzer, Nieke Knoben, Mari Laakso, Julia Lorke, Greg Mannion, Luciano Massetti, Alice Mauchline, Kai Pata, Andy Ruck, Pavel Taraba, Silvia Winter

**Affiliations:** ^1^Trinity College Dublin, Dublin, Ireland; ^2^University of Lisbon, Lisbon, Portugal; ^3^University of Sydney, Sydney, NSW, Australia; ^4^University of Neuchâtel, Neuchâtel, Switzerland; ^5^Naturalis Biodiversity Centre, Leiden, Netherlands; ^6^Ministry of Agriculture and Forestry, Helsinki, Finland; ^7^Wissenschaft im Dialog, Bürger Schaffen Wissen, Berlin, Germany; ^8^University of Stirling, Stirling, United Kingdom; ^9^Institute of Bioeconomy, Italian National Research Council, Rome, Italy; ^10^University of Reading, Reading, United Kingdom; ^11^Tallinn University, Tallinn, Estonia; ^12^University of the Highlands and Islands, Perth, United Kingdom; ^13^Tomas Bata University in Zlín, Zlín, Czechia; ^14^Vienna University of Natural Resources and Life Sciences, Vienna, Austria

**Keywords:** learning environments, teachers, ontology and epistemology, activism, science communication, public engagement

## Abstract

Citizen science is a growing field of research and practice, generating new knowledge and understanding through the collaboration of citizens in scientific research. As the field expands, it is becoming increasingly important to consider its potential to foster education and learning opportunities. Although progress has been made to support learning in citizen science projects, as well as to facilitate citizen science in formal and informal learning environments, challenges still arise. This paper identifies a number of dilemmas facing the field—from competing scientific goals and learning outcomes, differing underlying ontologies and epistemologies, diverging communication strategies, to clashing values around advocacy and activism. Although such challenges can become barriers to the successful integration of citizen science into mainstream education systems, they also serve as signposts for possible synergies and opportunities. One of the key emerging recommendations is to align educational learning outcomes with citizen science project goals at the planning stage of the project using co-creation approaches to ensure issues of accessibility and inclusivity are paramount throughout the design and implementation of every project. Only then can citizen science realise its true potential to empower citizens to take ownership of their own science education and learning.

## Introduction

Citizen science has long been considered to hold vast potential in the field of science education and learning (Bonney et al., 2009a). It is also a rapidly growing field of research in its own right, with increasing prominence in areas such as astronomy, ecology, meteorology, and medicine (Lewandowski et al., 2017). As the term “citizen science” applies to science that involves people who are not professional scientists, it occupies a unique position in the scientific community. As well as being its own distinct field of enquiry (Jordan et al., 2015), it can also reach beyond individual scientific disciplines to attract wider public participation in scientific research, leading to the overall advancement of scientific knowledge (Bonney et al., 2009b). Citizen science has ample capacity for transdisciplinarity and for integrating natural, physical, and health sciences with the humanities and social sciences (Pykett et al., 2020; Tauginiene et al., 2020). It is an excellent method of harnessing non-traditional data sources to tackle societal challenges and contribute to certain Sustainable Development Goals of the United Nations (Fritz et al., 2019; Fraisl et al., 2020).

A number of associations have been established world-wide, with the aim of bringing together people who are involved in citizen science. The most distinguished of these are the Citizen Science Association (ostensibly a US-based association, but offering global membership), the European Citizen Science Association, and the Australian Citizen Science Association. Each of these relatively new associations have highlighted education and learning as critical issues for citizen science as an emerging professional field (Storksdieck et al., 2016; Roche and Davis, 2017a). Citizen science has the capacity to “develop connections between students' everyday lives and science so that they will have tangible reasons for continuing with the lifelong learning of science” (Jenkins, 2011, p. 501). It can function as a means of engaging the public with science on the scale of individual experiments, creating a unique position of combining participation, monitoring, and social change (Doyle et al., 2019; Dawson et al., 2020). Citizen science also offers a route by which the tenets of responsible research and innovation (Owen et al., 2012) may be fulfilled, particularly by facilitating lesser-heard communities in having their voices heard in relation to scientific policy-making and governance. This is now of more importance than ever, as researchers and academic experts find society's trust in their authority diminished (Roche and Davis, 2017b), while the COVID-19 pandemic has demonstrated the acute need for public trust to be strengthened (Henderson et al., 2020). Indeed, citizen science might offer a more pressing model for science in a post-pandemic world (Provenzi and Barello, 2020). Despite its growing importance, citizen science is rarely considered in terms of science education research (Kelemen-Finan et al., 2018). For the purposes of this paper, the term “science” is taken to encompass systematic and evidence-based investigations in the pursuit of new knowledge, while the term “education” is considered to be the acquisition of knowledge through learning. Learning can be self-directed, but often relies on the guidance of a teacher. The learning can take place in either formal or informal environments and the methods of teaching, or pedagogy, can be as varied as the settings themselves. While these definitions are not all-inclusive, they provide a starting point where science, education, and learning can be considered in relation to the emerging challenges and opportunities stemming from citizen science.

### Supporting Citizen Science in Education and Learning

In order to ensure that citizen science lives up to its vast potential to extend beyond individual projects and disciplines, opportunities for strengthening the relationship between citizen science and education must be identified so that appropriate support can be offered and integration achieved. Many questions have been raised regarding the role of citizen science within science education (Bonney et al., 2016); even the term “citizen science,” and the individual component words of that term can have different meanings subject to context (Eitzel et al., 2017). A report prepared by the US National Academies of Sciences, Engineering, and Medicine set out to tackle these issues—not simply by discussing the “potential of citizen science to support science learning” but by endeavouring to “identify promising practices and programs that exemplify the promising practices, and lay out a research agenda that can fill gaps in the current understanding of how citizen science can support science learning and enhance science education” (National Academies of Sciences, 2018, p. 2).

In Europe, efforts are also underway to identify the challenges and opportunities that may arise when citizen science and education are brought together through project collaboration, networks, research, and practice. Alongside the European Commission's “Science with and for Society” work programme (European Commission, 2017), and the European Citizen Science Association, a COST Action (CA15212) was also established in order “to promote creativity, scientific literacy, and innovation throughout Europe” through citizen science (COST, 2016). This COST Action included a working group entitled “Develop synergies with education” and, through a dedicated workshop, brought together researchers and practitioners with a range of different backgrounds and contexts for interpreting citizen science in relation to education and learning. The subsequent discussions that emerged from the working group led to this paper, which provides an international perspective on some of the main challenges and opportunities facing citizen science in education. While the diversity of the working group ensured that a broad selection of perspectives were considered, it was by no means exhaustive. There are undoubtedly other arising challenges and opportunities that have not yet been considered, and it is hoped that this paper will serve as a starting point for developing a comprehensive research agenda for supporting citizen science in education and learning.

## Challenges for Citizen Science, Education, and Learning

Every person who participates in citizen science is also involved in a learning process (Bela et al., 2016), not just the acquisition of the skills necessary for participation in citizen science, but also a deeper understanding of scientific concepts and processes—historically referred to as “scientific literacy” (Miller, 1983). The development of scientific literacy in tandem with the contribution to genuine scientific outcomes has been a longstanding goal of the field (Brossard et al., 2005; Jordan et al., 2011; Saunders et al., 2018). Logistical tensions tend to arise between citizen science and education due to unavoidable constraints concerning time, space, staff, and other key resources. While training could help address a number of these issues, the associated costs often present a barrier, especially in fields where participant goodwill and volunteer work are crucial (Lorke et al., 2019). Many citizen science projects have little flexibility in terms of timing and the allocation of resources, and navigating these issues will invariably remain challenging for many citizen science coordinators and programme managers.

Beyond logistics, the goal of citizen science—to bring about scientific progress—and the goal of education—to support learning—may not necessarily always align. Citizen science can be integrated into education in both formal and informal learning environments. Formal learning generally occurs in school, college, or university environments with clear learning objectives, whereas informal education can take place outside of the classroom or after school, often in public engagement spaces like museums, zoos, or aquariums (Eshach, 2007; National Research Council, 2009). Each environment raises unique challenges for practitioners. Challenges may also arise as a result of the different needs of the scientists, students, teachers, educators, researchers, and other actors involved; the issue of how information is communicated and shared; as well as from potential conflict between the capacity of citizen science for activism and the desire or obligation to reach specific learning objectives.

### Citizen Science and Education in Formal Learning Environments

Specific learning objectives, background information, and lesson plans are generally utilised by educators to integrate citizen science projects into curricula in formal learning environments, particularly when teaching children and adolescents at primary and post-primary level (Bonney et al., 2009b, 2014). Consequently, project engagement becomes contingent on the educators themselves; as the students or learners may have been effectively volunteered to participate, rather than electing to do so, motivation and engagement may be lacking compared to other groups of citizen science participants. Therefore teachers, as the citizen science intermediaries in formal learning environments (Weinstein, 2012), play a crucial role in successfully integrating such projects into their classrooms and schools. That some teachers may lack confidence in their own general level of scientific content knowledge and scientific literacy can considerably impede this process—for example, issues of content knowledge could arise on projects that require teachers to explore outdoor environments where they cannot fulfil the perceived demand to be an expert (Kelemen-Finan and Dedova, 2014; Jenkins et al., 2015). The participation of schools can also be constrained by school curricula, timetables, or logistical issues. For those teachers and schools that are interested in engaging in citizen science projects, it may be difficult to navigate the rapidly growing number of initiatives available to them.

Additional challenges stem from the type of classroom involvement that can be facilitated. Projects such as the Monarch Larva Monitoring Project (Kountoupes and Oberhauser, 2008) or Classroom FeederWatch (Bonney and Dhondt, 1997) are considered examples of best practice from the last two decades, where materials are provided for local school involvement, while generating valuable data for the project at large. Both of these projects offer web tools for downloading data, as well as instructions for data analysis to empower participants to perform their own analysis. The construction of materials, the maintenance of an interactive website, smartphone apps, and continuous email contact requires considerable resources, especially in terms of staff with relevant experience in science and education. More recent projects like the School of Ants (Lucky et al., 2014), LandSense (Olteanu-Raimond et al., 2018), and eMammal (Schuttler et al., 2019) have mirrored the success of these large-scale schools projects, while national schools-based citizen science projects in the future are likely to tackle aspects of post-pandemic life (Eichler et al., 2020; Ugolini et al., 2020). Smaller contributory projects sometimes lack such infrastructure and resources and, consequently, participants are often only involved in data collection without gaining experience of the complete inquiry process (Jenkins et al., 2015). Zoellick et al. (2012) argue that a third party, for example, a university, is a necessary intermediary between scientists and educators in order to ensure that specific research and educational outcomes are ultimately achieved. Their proposed model for school-based research projects describes scientists' and educators' inputs, their interactions during the design and implementation phase, and separate outputs and outcomes for students and scientists. While this model addresses the tension between collaborating scientists, schools, teachers, and students, it could be further improved with the added consideration of student input alongside outcomes for the educators (Jenkins, 2006). Co-constructed citizen science projects, where students are actively involved in the scientific process are labour and resource intensive for scientists, students, and teachers, but are more likely to achieve the scientific and educational goals of the project (Gray et al., 2012).

### Citizen Science and Education in Informal Learning Environments

Informal education generally refers to the learning that takes places outside of classrooms and lecture theatres. Informal environments may sometimes be further subdivided into non-formal and semiformal categories (Werquin, 2007), but for the purpose of this paper, all learning environments outside of those involving schools, higher education, or universities, can be considered informal. Informal learning environments, such as science centres and museums, are critical to science education. Citizen science projects find a natural home in these domains due to a shared strong commitment to public engagement (Dickinson et al., 2012; Ballard et al., 2017).

The impact that citizen science projects can have on education in these environments is affected by the same challenge that faces informal learning environments in general—finding the best way to support learners and facilitators (Stewart and Jordan, 2017). Tension may arise between designing projects that are “fun” for casual participants and ensuring that data generated is of sufficient quality. The use of “fun” activities can increase participation, create interest in a given research topic, and nurture a love of science—particularly in projects involving young people (Kountoupes and Oberhauser, 2008). However, there may be a trade-off regarding the time and resources necessary to make these activities engaging, and the efforts to serve the scientific and educational goals of the project. A report by the US Committee on Learning Science in Informal Environments in 2009 found that although tensions often arise between the “reasonable goals for learning science in informal environments” and the education “agenda,” it was deemed ‘unproductive to blindly adopt either purely academic goals or purely subjective learning goals' in informal learning settings (Bell et al., 2009, p. 3).

The learning that takes place in informal environments through citizen science projects can be difficult to capture. Initial efforts have been undertaken to find ways to evaluate the intended learning outcomes for the participants in these projects (Phillips et al., 2014, 2018), but Edwards (2014) has highlighted that the specific impact that citizen science can have on the lifelong learning of people outside the classroom has not yet been comprehensively explored. Likewise, while understanding social and cultural capital is critical to interpreting how people engage with informal science education institutions (Dawson, 2014), there has not yet been enough consideration given to how this capital affects participation in citizen science projects and the resulting issues of equity that may emerge (Birmingham, 2016). Citizen science has the same issues of inequity that are endemic throughout society, with innate barriers to participation for minorities and underserved communities (Soleri et al., 2016; Fiske et al., 2019). Science capital—a concept that explores how a person's environment and social class can affect their involvement in science—could allow “inequalities in science participation” to be discovered more readily, which in turn could be used to promote “social justice within science education” (Archer et al., 2015, p. 943). If citizen science is to fulfil its potential in improving equity of access to, and participation in, both science and science education in informal learning environments, then “the extent to which citizen science can build science capital and enable wider engagement with science-related issues [...] deserves further experimentation and investigation” (Edwards et al., 2018, p. 390).

### Citizen Science, Education, and Activism

Arnstein (1969) pioneered the concept of citizen participation with her “Ladder of Citizen Participation,” which described the eight levels of citizen power, from non-participatory “manipulation” to “citizen control.” The role citizen science may play in activism and in advocacy—citizens intervening on behalf of, or representing, a socio-political goal (Letiecq and Anderson, 2017; Reis, 2020)—is a key consideration in its interactions with education and learning. From the perspective of civic society, citizen science should encourage individuals to take an active role in their communities—operationalizing active citizenship (Burls and Recknagel, 2013). This role of active citizenship aligns with Arnstein's rising level of citizen participation and is especially pertinent in citizen science projects that focus on environmental activism and climate change—empowering people to take responsibility for the future of their environments (Baptista et al., 2018; Kythreotis et al., 2019; Dawson et al., 2020). The concept of active citizenship is closely aligned to the UNESCO Incheon Declaration and Framework for Action (UNESCO, 2015) which seeks to ensure inclusive, equitable, and quality education on a global scale. It encompasses three distinct dimensions: a citizen's legal citizenship, socio-economic background, and socio-cultural background (Kalekin-Fishman et al., 2007). Legal citizenship enables an individual to channel their political agency, although, as highlighted by Eitzel et al. (2017), the definition of citizenship is complex and can be problematic in some contexts. Socio-economic power can create demand for education, transforming learning into a desirable consumer commodity and potentially creating resources that can supplement underfunded or overlooked government services. The socio-cultural dimension of active citizenship focuses on ethics, and seeks to foster cohesion, inclusion, and tolerance in the personal and public spheres. Citizen science practice could be exercised as one means of educating active citizens; by empowering communities to advocate for their local environment through research, or by enabling citizens to gather evidence on, and articulate, pressing issues. The results of active citizenship, often shared with the wider public through social media, can even hasten the actions of decision-makers (Eitzel et al., 2017).

However, despite the benefits of potentially bolstering science education through active citizenship, tension may arise between the traditional role of the learner in some learning environments, acquiring pre-determined knowledge and values, and the process of learning continuously through active citizenship, which may result in social transformation. Educators may feel uncomfortable in sharing decision-making power with other participants in citizen-led activities and may feel uncertainty as to the value of that learning process (Mueller and Tippins, 2015). In citizen science activities, practitioners, and participants may not be able to retain their usual roles in some learning environments (Fazio and Karrow, 2015) and significant changes may need to be made in order to enable and facilitate social activism.

### Theoretical Perspectives on Citizen Science and Educational Practice

Ontologies and epistemologies are theories surrounding the nature of being and knowing, or generating knowledge, and provide the assumptions which naturally underlie both educational practice and citizen science practice. Ontology and epistemology are often linked, because how the world is understood, and the phenomena that are available for study within it, are very much dependent on how people think they can come to know, and what they consider “valid” knowledge. Therefore, onto-epistemological differences, namely, tensions that arise from the disparate ways each person interprets the world, including the understanding of what phenomena can be studied, how it can be studied, and the conclusions that can subsequently be drawn, mean that the differences inherent between various citizen science fields and educational environments will result in disparate learning outcomes. As noted by Shirk et al. (2012), tension may be generated due to the often dissimilar interests of scientific and public stakeholder groups in the wider field of public participation in scientific research (PPSR), in which citizen science is intrinsic. Competing onto-epistemologies are likely contributory factors to the difficulties inherent in engaging various publics in scientific research, and the alignment of these competing constituents could facilitate greater synergy between citizen science and education.

Building on Arnstein's concept, Haklay (2013) designed an adapted model for citizen science in which the fourth and final level of citizen participation enables all stakeholders—scientists, educators, facilitators, the public, education partner organisations, and policy makers—to collaborate. At this level, citizen science would emerge as a truly transformative practice that has the power to change and influence the world. In his typology, Haklay's (2013) suggests that increasing the involvement and engagement of the public in citizen science will result in the empowerment of learners while significantly democratising citizen science input. As members of the public are empowered to engage more deeply with, and learn more about the scientific projects they are involved in, they are likely to move up the structure—from merely acting as sensors for science projects that are conducted elsewhere, to collaboratively shaping scientific endeavours from their inception, and participating in their analysis throughout.

Competing tensions in citizen science can also be considered through three stances in education suggested by Stetsenko's (2008) acquisition, participation, and transformation—which are evident at each level of Haklay's typology. In the first stance, “acquisition,” stakeholders see citizen science processes as being concerned with generating pre-existing, fixed, factual knowledge that is gained by individuals primarily through passive input. The second stance, “participation,” positions science and education practices as potentially being affected by other factors—such as location or culture—and necessitates an initiation process in order for participants to gain full access to the community. This stance places citizen scientists into a more participatory role, and educators and scientists are aware that citizen science often generates findings that are culturally located, generated, shared, mutable, and communicated over time. This stance may bring about tension from stakeholders who don't wholly subscribe to the idea that findings are culturally embedded; however, “participation” provides access for novices, e.g., pupils, into the community of science practitioners.

Applying Stetsenko's third stance, citizen science can become “transformative” when embedded in educational programming. This transformation could lead to change at individual, community-wide, and global levels if citizen science expands in scale and scope. The intrinsic risk of the transformative approach is that it can replace a system of knowledge with one that still does not appropriately recognise marginalised forms of knowledge (Leibowitz, 2017). An example of a transformative project could be “WeatherBlur” a co-created citizen science project bringing together, fishermen, students, teachers, and research scientists from island and coastal communities on the east coast of the US “to share, analyse, and interpret data about the local impact of climate change” (Kermish-Allen et al., 2019, p. 627). “Knowing” and acquiring knowledge are presented by Stetsenko as active and collective activities; thus citizen science would evolve into a collaborative, co-creative approach. This transformative stance embodies the fourth level of Haklay's typology; presenting an ideal common ground for both education and citizen science, resolving potential onto-epistemological tensions, and generating synergy.

### Dissemination, Dialogue, and Participatory Communication

Citizen science projects often aim not only to advance scientific knowledge, but to share it too. The manner in which communication takes place in these projects, and the effect it has on learning, must tread the line between outreach and engagement, and warrants a communication plan that not only connects with the right audiences but retains their interest over time (Veeckman et al., 2019). Projects tend to adopt either a two-way approach that emphasises participatory dialogue (McCallie et al., 2009; Haywood and Besley, 2013), or a one-way approach that focuses on outreach and dissemination.

Two-way communication between citizens and scientists within projects leads to the sharing of ideas, information, and knowledge, while one-way dissemination to a wider audience can involve the communication of results, funding-specific public relations obligations, or participant recruitment (Tulloch et al., 2013; Groulx et al., 2017). While the two-way participatory approach is more time consuming, and can put additional pressure on project resources, it is more likely to foster collaborative work, relationship building, and learning (Mercer and Littleton, 2007). The tension between outreach and engagement is mirrored in the field of science communication with its models of deficit and dialogue (Trench, 2008; Lewenstein, 2015).

Whereas participatory engagement is a powerful way to support learning (Gleason and Von Gillern, 2018), one-way dissemination also has a valuable role in citizen science. Communicating the mission and vision of a project outside of its immediate community can be one of the most important goals for project leaders (Kerzner, 2013). The way in which these values are communicated can vary, depending on the scientists, citizens, and policymakers involved. In particular, there is often a perceived disconnect between policymakers and other key stakeholders, such as citizens and scientists (Socientize Consortium, 2013). Using a Public Relations (PR) approach is a commonly employed method of bridging this gap (Scott, 2013), and involves implementing a strategic communications plan that can include public lectures, workshops, festivals, exhibits, tours, and open laboratories. To supplement these activities, a strategic PR plan for citizen science projects is often used to directly engage policymakers with demonstrations of the usefulness of the project and the need for new knowledge generation (Socientize Consortium, 2014). Although a common concern when employing a communication approach that focuses on PR is the potential tendency to overlook negative results and issues of uncertainty, which are part of the scientific process, if effective communication is adhered to between stakeholders, it can lead to citizen science projects enhancing public debate and citizen participation in decision-making processes, especially regarding societal challenges (Newman et al., 2012).

In as much as onto-epistemological tensions may arise between citizen science and education, one-way dissemination may generate significant tension in a learning environment when science is positioned as the sole truth, and the scientific method the only way to produce reliable knowledge. A two-way participatory approach, by contrast, not only bridges the gap between science education and science communication but poses science as one of many types of knowledge, and the scientific method as one of a multitude of ways to describe the world (Baram-Tsabari and Osborne, 2015). This interplay between science and society is ever more critical in the era of fake news and misinformation (Scheufele and Krause, 2019). One of the most effective solutions to such tensions is to involve scientists in all aspects of the communication process in citizen science projects (Riesch and Potter, 2014). This has a positive effect on participant recruitment, retention, instruction, knowledge sharing, awareness raising, and increases the credibility and authority of the work taking place. However, some scientists may be hesitant to engage in efforts to communicate if they feel that they are not specifically trained to do so (Golumbic et al., 2017). Communication activities, such as public talks, interviews, or popular science articles can be time consuming, and some scientists may find participation uncomfortable (Van Vliet et al., 2014). An increasing number of research funding initiatives at both national and European levels require the inclusion of public engagement and communication strategies, thus increasing the pressure on scientists involved in citizen science projects to directly engage with public audiences. This may be particularly challenging for scientists if these activities are not supported by their institutions, or if their career progression is primarily evaluated on the quality of their publications in scientific journals (Kreiman and Maunsell, 2011). While not without its critics (Khazragui and Hudson, 2015; Watermeyer, 2016), the Research Excellence Framework in the UK is a notable example of a research evaluation process that gives consideration to the societal impact of research.

## Opportunities for Citizen Science, Education, and Learning

Thoroughly exploring the obstacles that prevent the successful integration of citizen science practice into mainstream education systems is key to overcoming them. Recommendations based on the challenges that have been highlighted in this paper could help citizen science to fulfil its potential as a truly transformative social innovation for education and learning. This could, in turn, encourage citizen science practitioners and educators to take an adaptive and flexible position in the face of new and emerging societal challenges and a fluctuating political and economic landscape that continuously strains the relationship between science and society.

### Recommendations for Finding Synergy

There has already been a great deal of work conducted with a view to establishing best practice principles for citizen science notably, the European Citizen Science Association's “Ten Principles of Citizen Science” [European Citizen Science Association, 2015; and the subsequent characteristics of citizen science (European Citizen Science Association, 2020) which expand on the principles]. Assuming adherence to these principles, the following recommendations may create meaningful opportunities for citizen science in education and learning.

Professional development training workshops (Jeanpierre et al., 2005) facilitating citizen science in classrooms can be effective in overcoming some of the barriers that schools, teachers, and students may encounter while participating in citizen science projects (Eberbach and Crowley, 2009; Scheuch et al., 2018). Crall et al. (2013) demonstrated that such workshops could improve scientific literacy for workshop participants, assessed with context-specific measures. However, unique challenges are still likely to arise. Jordan et al. (2011) could not detect any increase in scientific literacy, and the potential failure of these training sessions was attributed to a lack of time for active learning, which must provide a provision for reflection, and allow participants to make mistakes (Gray et al., 2012; Jordan et al., 2015). To further embed citizen science in informal learning spaces, gamification is an effective tool in engaging participants, and in-game rewards can be carefully planned in order to reward focus on good quality data (Tippins and Jensen, 2012; Bowser et al., 2013; Morschheuser et al., 2019; Piper, 2020).

Ensuring alignment between the onto-epistemological positions of the citizen science, education, and learning aspects of any project is a worthwhile endeavour. It is clear that the achievement of the educational goals of citizen science projects are contingent on those goals being taken into consideration at the design stage (Bonney et al., 2014). Following frameworks for measuring individual learning outcomes from participation in citizen science—such as Phillips et al. (2018)—would facilitate the alignment of learning outcomes and the underlying onto-epistemological stances. Additionally, building a co-creation component into citizen science projects from the outset would significantly increase the likelihood that both the educational and scientific goals of the project will be met (Gray et al., 2012). Such co-creation approaches should be considered obligatory, where possible, for every new project.

Challenges surrounding communication, dissemination, and dialogue may be addressed by increasing science communication training opportunities for scientists involved in citizen science, as well as for scientists in general. Collaboration between scientists and citizens with public relations and communication professionals could lead to more open strategies for communicating with different audiences and could generate clear alignment between both the dissemination and participatory modes of communication. Crucially, to ensure that scientists contribute not only to the scientific goals of citizen science projects, but also to the communication and educational aspects, public engagement should be recognised as scholarly work. This would be made possible through research institutions redefining public engagement as a metric to be evaluated in academic career progression, in a manner akin to research output (Smith et al., 2014).

The greater recognition of citizen science and activism in recent years may, in part, be due to a growing focus on equality, open access, and public participation caused by the current global political climate (Roche and Davis, 2017b; Hutter and Kriesi, 2019). Once public engagement is fully integrated into the missions of both research performing organisations and research funding organisations, social activism must be given due consideration as an inevitable counterpart to citizen science. As recommended by the National Academies of Sciences (2018), issues of equity and power should be given particular consideration at all stages of citizen science project design and implementation, in all settings. Citizen science is not merely a method of involving the public in scientific research but is also a way of empowering citizens to take ownership of their own science education and learning.

### The Future of Citizen Science in Education and Learning

Transformative approaches to education are becoming more widely accepted; within education, and in higher education specifically, there is significant interest in developing co-researcher partnerships (Healey et al., 2016). Such partnerships can lead to the co-design of curricula (Bovill, 2014) and the co-production of knowledge (McCulloch, 2009). A contributory approach necessitates a whole new learning paradigm requiring novel educational methods. The outdated metaphor of ‘students as consumers' (Dearing, 1997; Palfreyman and Warner, 1998), which has a negative impact on student engagement and performance (Bunce et al., 2017), could be replaced by a citizen science partnership that supports educators and students, where knowledge is exchanged in both directions, and the students are active partners in their learning (Freeman et al., 2014) and in participating in authentic scientific research.

Citizen science practitioners and programmes seeking links with schools may find that tapping into more transformative models of learner engagement is a starting point for enhanced participation. The adoption of a transformative onto-epistemological stance opens up much greater potential for synergy between citizen science and education. The outcomes of transformative citizen science will result in changes to what is known, how it is known, and to the individual, socio-cultural, and wider world. Mueller and Tippins (2012) rhetorically ask why citizen science programming in education generally aims to advance science literacy, when learners' motivations are predominantly to care for what is often a local environment. Within this transformational framing, potential exists for attending to learning and practicing science in ways that are more in tune with learners' motivations, with local places, and in ways that are socio-culturally distributed among all participants, including scientists, teachers, students, community members, policymakers, and any other stakeholders (Mannion et al., 2013; Haywood et al., 2016). Taking a transformative stance on citizen science in education could be key to engendering a more vital role for science in the public sphere, generating responses to current and future eco-social problems (Dillon et al., 2016), and helping to achieve the UN Sustainable Development Goals (Fritz et al., 2019; Fraisl et al., 2020).

The future of how citizen science will be integrated into education and learning will continue to be influenced by globally-accessible digital platforms. The newest of these, EU-Citizen.Science, is an online platform for citizen science in Europe that is being established with the support of a Horizon 2020 grant from the European Commission. This platform will not only make citizen science projects and data more readily accessible, but it will also act as a mutual learning space for sharing useful tools, guidelines, training, and best practice examples in several languages to help citizens, scientists, teachers, students, schools, and other stakeholders to determine how they can engage with local and international citizen science projects. Global initiatives such as these will be key to realising the education and learning potential of citizen science as a far-reaching social innovation.

## Author Contributions

JR and SW were the lead authors and oversaw the completion of the writing. JR, LB, CG, YG, LK, NK, ML, JL, GM, LM, AM, KP, AR, PT, and SW contributed writing to individual subsections of the manuscript. All authors have read and agreed to the published version of the manuscript and were involved in the conceptual design of the manuscript.

## Conflict of Interest

The authors declare that the research was conducted in the absence of any commercial or financial relationships that could be construed as a potential conflict of interest.
